# Integrating Embryonic Development and Evolutionary History to Characterize Tentacle-Specific Cell Types in a Ctenophore

**DOI:** 10.1093/molbev/msy171

**Published:** 2018-08-30

**Authors:** Leslie S Babonis, Melissa B DeBiasse, Warren R Francis, Lynne M Christianson, Anthony G Moss, Steven H D Haddock, Mark Q Martindale, Joseph F Ryan

**Affiliations:** 1 Whitney Laboratory for Marine Bioscience, University of Florida, St. Augustine, FL; 2 Monterey Bay Aquarium Research Institute (MBARI), Moss Landing, CA; 3 Department of Biological Sciences, Auburn University, Auburn, AL

**Keywords:** trait loss, comparative transcriptomics, ctenophore, novelty, development, biological adhesive

## Abstract

The origin of novel traits can promote expansion into new niches and drive speciation. Ctenophores (comb jellies) are unified by their possession of a novel cell type: the colloblast, an adhesive cell found only in the tentacles. Although colloblast-laden tentacles are fundamental for prey capture among ctenophores, some species have tentacles lacking colloblasts and others have lost their tentacles completely. We used transcriptomes from 36 ctenophore species to identify gene losses that occurred specifically in lineages lacking colloblasts and tentacles. We cross-referenced these colloblast- and tentacle-specific candidate genes with temporal RNA-Seq during embryogenesis in *Mnemiopsis leidyi* and found that both sets of candidates are preferentially expressed during tentacle morphogenesis. We also demonstrate significant upregulation of candidates from both data sets in the tentacle bulb of adults. Both sets of candidates were enriched for an N-terminal signal peptide and protein domains associated with secretion; among tentacle candidates we also identified orthologs of cnidarian toxin proteins, presenting tantalizing evidence that ctenophore tentacles may secrete toxins along with their adhesive. Finally, using cell lineage tracing, we demonstrate that colloblasts and neurons share a common progenitor, suggesting the evolution of colloblasts involved co-option of a neurosecretory gene regulatory network. Together these data offer an initial glimpse into the genetic architecture underlying ctenophore cell-type diversity.

## Background

**Fig. 1.**
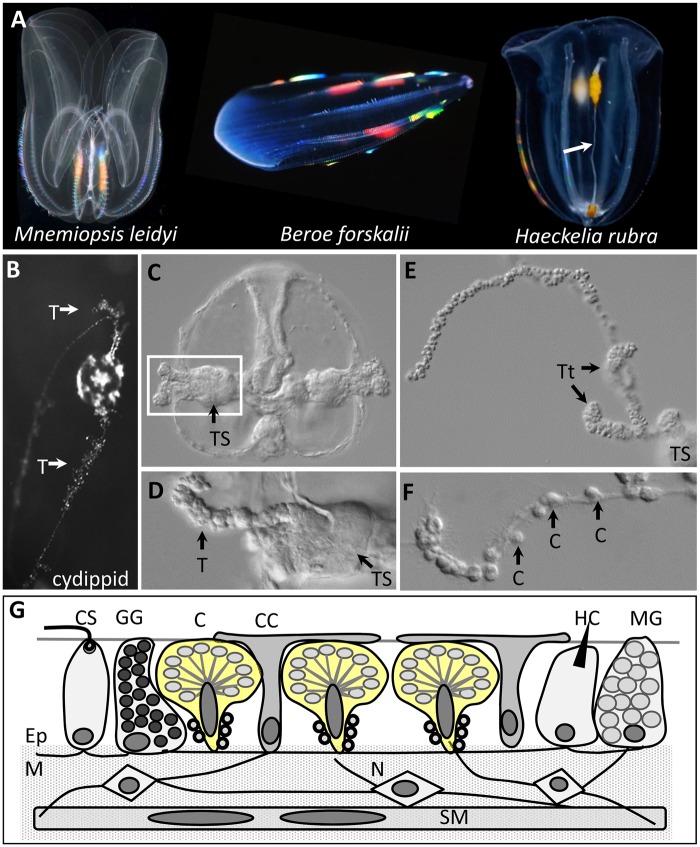
Tentacle morphology varies across ctenophores. (*A*) *Mnemiopsis leidyi* has reduced tentacles as an adult, *Beroe forskalii* lacks tentacles completely, and *Haeckelia rubra* has long unbranched tentacles (white arrow). (*B*) The cydippid (larval) stage of *M. leidyi* has long branched tentacles (T). (*C*) DIC micrograph of a cydippid with tentacles retracted into the tentacle sheath (TS). (*D*) Higher magnification of the boxed area in C showing a branched tentacle (T) emerging from the tentacle sheath. (*E*) Emerging tentacle showing partially contracted side branches (tentilla, Tt). (*F*) Fully extended tentacle showing colloblast islets (C) along the tentacle. (*G*) Tentacle cell types—neurons (N) and smooth muscle cells (SM) are in the mesogleal core (M); colloblasts (C; yellow), covering cells (CC), ciliated sensory cells (CS), granular gland cells (GG), hoplocytes (HC), and mucous gland cells (MG) are in the epidermis (Ep). Images in panel *A* courtesy of Bruno Vellutini (*M. leidyi*) and Steve Haddock (*B. forskalii*, and *H. rubra*).

Insight into how novelty is generated is important for understanding the origin and diversification of multicellular life. An outstanding challenge, however, is finding a model for which the direction of evolutionary change is known and the novelty of interest is easy to characterize. Ctenophores (comb jellies) are gelatinous marine invertebrates that diverged from the rest of animals over 800 Ma (Dohrmann and Worheide 2017); although their phylogenetic position remains contentious (Dunn et al. 2008; Ryan et al. 2013; Moroz et al. 2014; Borowiec et al. 2015; Simion et al. 2017; Whelan et al. 2017), they are clearly among the first lineages to diverge from the rest of animals. While they share several anatomical features in common with bilaterian animals (e.g., neurons and muscle cells), ctenophores are defined by two novel traits: parallel rows of cilia organized into “combs,” and colloblasts, the adhesive cells used to capture prey (fig. 1). Found exclusively in the tentacles, colloblasts are typified by a crown of adhesive-filled secretory vesicles and an extensible basal apparatus (Eeckhaut et al. 1997). Upon contact with prey, the apical membrane of the colloblast ruptures, releasing the adhesive (Franc 1978). Their association with tentacles and their specialized role in prey capture have led some to propose that colloblasts are the functional analogs of the cnidarian cnidocyte (stinging cell; Alie et al. 2011; Borisenko and Ereskovsky 2013).

The tentacles of ctenophores are composed of a central axis of muscle and nerve fibers embedded in a gelatinous extracellular layer (the mesoglea) surrounded by a monolayer of epidermal cells. In many (but not all) species of ctenophore, the tentacles are adorned by numerous side branches (tentilla) and in some ctenophores (e.g., *Euplokamis*), these side branches are extensible and prehensile (Mackie et al. 1988). During feeding, the tentacles and tentilla (when present) are extended or uncoiled into the water column to ensnare passing prey (Mackie et al. 1988; Emson and Whitfield 1991). While colloblasts have been described as the predominant cell type of the tentacle/tentillum epidermis, several other cell types are known to populate these tissues (fig. 1*G*): covering cells, also known as cap cells or support cells, two types of sensory neurons (ciliated sensory cells and hoplocytes/peg cells), and two types of gland cells (mucus-secreting and granular gland cells; Horridge 1965; Emson and Whitfield 1991; Eeckhaut et al. 1997; Borisenko and Ereskovsky 2013; Carre and Carre 1989).

The feeding behaviors of ctenophores are diverse but typically involve entangling prey in the extended tentacles or trapping prey with the oral lobes (Haddock 2007). One group of ctenophores (genus *Haeckelia*) has tentacles devoid of colloblasts; instead, their tentacles are populated by cnidocytes sequestered from their cnidarian prey (Carre and Carre 1980; Mills and Miller 1984). Lacking tentacles completely, ctenophores in the genus *Beroe* (the sister group to *Haeckelia*; Podar et al. 2001; Simion et al. 2015) engulf their prey (other ctenophores) with expanded lips and remove chunks of tissue using “teeth” made from modified cilia (Tamm 1983; Haddock 2007). Many species of lobate ctenophore (e.g., *Mnemiopsis leidyi*, *Bolinopsis infundibulum*) undergo ontogenetic change in their behavior, relying on the use of tentacles in the juvenile stage and oral lobes as adults. In these taxa, the adult tentacles are short and become restricted to an oral fringe following metamorphosis. In contrast, the adult *Beroe* develops directly from an atentaculate larva. Thus, whereas the gene regulatory network underlying the development of tentacles may be downregulated in the adult stage of many lobate species, this network may not function at any stage in beroids.

Although they are a clear example of an evolutionary novelty, little is known about the origin of colloblasts. In this study, we leveraged the evolutionary history of ctenophores (including phylogeny, genes loss, and trait loss) to identify genes specific to this novel cell type. We hypothesized that some of the genes associated with colloblast development would have been lost during the diversification of *Beroe* and *Haeckelia* from their colloblast-bearing ancestor. Likewise, we hypothesized that tentacle genes would have been lost in the stem lineage of *Beroe*. Using comparative transcriptomics, we searched for genes that were present in most ctenophores but were absent from lineages that lack colloblasts and tentacles. We tested the hypothesis that these were trait-specific genes by examining their expression during tentacle morphogenesis in *M. leidyi* using fine-scale temporal RNA-Seq. We further validated these results using adult tissue-specific and cell-specific RNA-Seq data sets. Using this approach, we report the first genetic characterization of the colloblasts, a truly novel and poorly understood cell type.

## Results

### Colloblasts Were Secondarily Lost from *Beroe* + *Haeckelia*

**Fig. 2.**
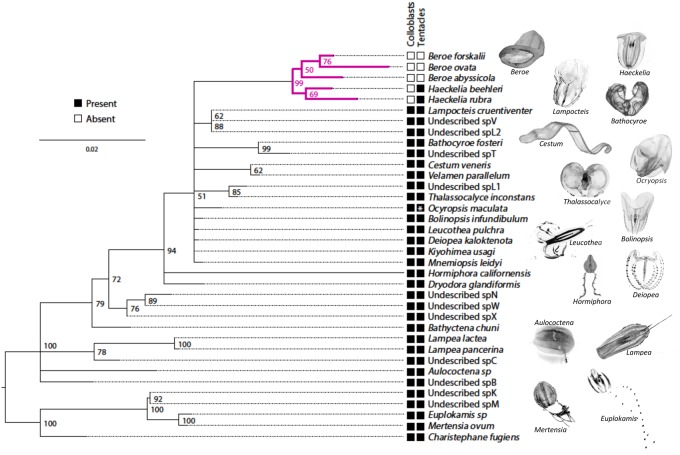
Beroe and *Haeckelia* are nested within Ctenophora. An 18S phylogeny of 36 species of ctenophore. Bootstrap support values are indicated at the nodes and branch length indicates rate of substitution. Clades with <50% bootstrap support have been collapsed. Shaded boxes indicate the presence of the indicated trait. *Indicates the presence of tentacles only in the larval stage.

We assembled a species tree using 18S sequences from 36 species of ctenophore (fig. 2). Our tree is congruent with previous reports of relationships among clades within Ctenophora (Podar et al. 2001; Simion et al. 2015; Whelan et al. 2017) and supports both the monophyly of the *Beroe* + *Haeckelia* clade and the position of this clade within the larger clade of colloblast-bearing lineages. This topology confirms that lack of colloblasts and lack of tentacles are derived traits.

### Identifying Colloblast and Tentacle Candidate Genes

**Fig. 3.**
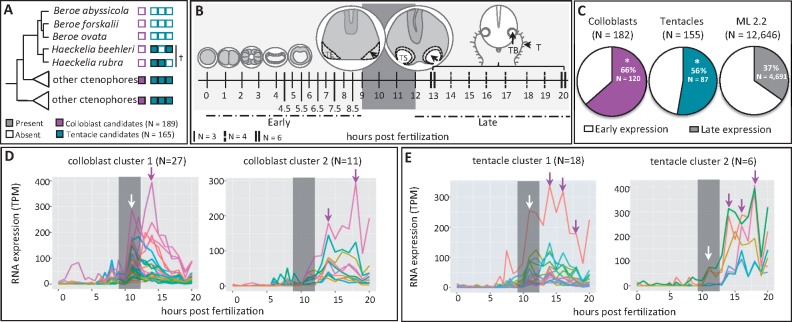
Identifying colloblast and tentacle candidate genes. (*A*) Summarized 18S tree. Colloblast candidates (*N* = 189) were present in ≥70% of the sampled taxa but absent from *Beroe* and *Haeckelia* (magenta boxes). Tentacle candidates (*N* = 165) were present ≥70% of the sampled taxa, including at least one species of *Haeckelia* (dagger), but were absent from *Beroe* (teal boxes). (*B*) Developmental transcriptome sampling for *Mnemiopsis leidyi*. Early cleavage occurs during the first 8 h post fertilization (hpf). A thickened tentacle epithelium (TE) is visible at 9 hpf and invagination of the epithelium to form the tentacle sheath (TS) is complete by 12 hpf. Larval tentacles (T) grow continuously from the tentacle bulb (TB). (*C*) Candidate data sets are enriched for genes expressed during tentacle morphogenesis, relative to gene models from *M. leidyi* (ML2.2) (*P* < 0.0001 for both). (*D*, *E*) The two largest clusters of gene expression profiles identified from candidate genes; colored lines represent different genes. Grey bars denote the 9–12 hpf window highlighted in panel *B*. White arrows indicate peaks in expression during tentacle bulb invagination, magenta arrows indicate peaks during tentacle morphogenesis. TPM—transcripts per million mapped reads.

To identify colloblast and tentacle candidate genes, we searched for genes that were missing from taxa lacking these traits. To do this, we sequenced and assembled transcriptomes from the same 36 taxa, including three species of *Beroe* and two species of *Haeckelia*. In most cases, transcriptomes were generated from adult animals; for *M. leidyi* and *Beroe ovata* transcriptomes were assembled from a combination of adults, embryos, and larvae. Using OrthoFinder (Emms and Kelly 2015), we generated 13,483 groups of orthologous genes, of which 189 contained representatives from at least 70% of all ctenophore taxa (including *M. leidyi*) but lacked *Beroe* and *Haeckelia*. Hereafter we refer to these as “colloblast candidate genes” (fig. 3*A*). Likewise, 165 groups contained orthologs from 70% of the taxa, including *M. leidyi* and at least one species of *Haeckelia*, but lacked *Beroe* (“tentacle candidate genes”). We confirmed that both sets of candidate genes were absent from the transcriptome and also the genome of *B. ovata* (European Nucleotide Archive accession number PRJEB23672).

We hypothesized that colloblast- and tentacle-specific genes would be expressed during or after the onset of tentacle outgrowth (Martindale 1986; Alie et al. 2011). To test this, we examined gene expression during the first 20 h of development in *M. leidyi* using an RNA-Seq time course (fig. 3*B*). After removing genes with no expression (7/189 colloblast genes and 10/165 tentacle genes), we found that 66% (120/182 genes) of the expressed colloblast candidates and 56% (87/155 genes) of the expressed tentacle candidates had higher abundance during tentacle morphogenesis (12–20 h post fertilization, hpf) than during early development (0–9 hpf; fig. 3*C*). We compared this to the number of *M. leidyi* protein models (ML2.2; https://research.nhgri.nih.gov/mnemiopsis/; last accessed September 10, 2018) which were expressed during this time course (12,646/16,548 models) and found that only 37% of the protein models (4,691/12,646 models) exhibited higher expression during tentacle morphogenesis. Using a random sampling approach (see Materials and Methods), we found that both sets of candidate genes were significantly enriched for late-expressed genes (*P* < 0.0001 for each).

Next, we used quality threshold (QT) clustering (Heyer et al. 1999) to group candidate genes with similar expression patterns. Among colloblast candidates, the two largest clusters consisted of 27 and 11 genes (fig. 3*D*). The cluster containing 27 genes was characterized by a peak in expression at 11 hpf followed by a second peak at 14 hpf whereas genes in the cluster containing 11 genes first peaked at 14 hpf with a second peak at 18 hpf. The two largest clusters of tentacle candidates consisted of 18 and 6 genes (fig. 3*E*). Both clusters exhibited an early peak at 11 hpf followed by peaks at 14 hpf, 16 hpf, and 18 hpf. (Accession numbers for clustered genes are provided in supplementary file 1, Supplementary Material online.)

**Fig. 4.**
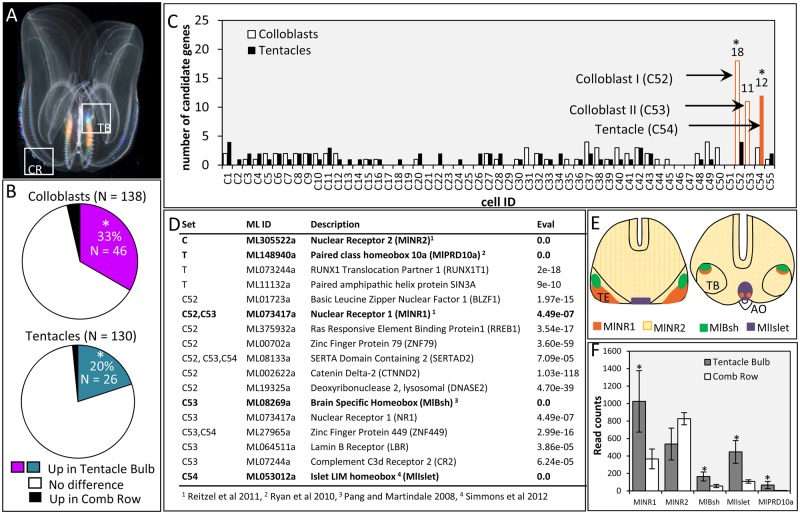
Colloblast and tentacle candidates are expressed together in adult *Mnemiopsis leidyi*. (*A*) Tissue sampling protocol for tentacle bulb (TB) and comb row (CR) transcriptome sequencing. (*B*) Over 70% of the colloblast and tentacle candidate genes were expressed in adult tissues (*N* = 138/189 colloblast candidates, *N* = 130/165 tentacle candidates). In both data sets, a significant proportion of the expressed genes (*P* < 0.0001 for both colloblast and tentacle candidates) were upregulated in the tentacle bulb, relative to the comb row (≥2 log_2_-fold change, padj < 0.05). (*C*) There was a significant cluster of colloblast candidates in cell C52 (*P* < 0.0001), with a second cluster in cell C53. Tentacle candidates clustered significantly in cell C54 (*P* = 0.0015). Cell IDs refer to single-cell sequencing results reported in Sebe-Pedros, Chomsky, et al. (2018). (*D*) Seventeen transcription factors were identified from colloblast candidates (C), tentacle candidates (T), or cells C52, C53, or C54 using GO terms GO: 0003677, GO: 0003700, GO: 0006351, and GO: 0006355. *E*-values represent the reciprocal best BLAST hit in the human genome, except for genes already identified from *M. leidyi* (Eval = 0.0). Only significant hits (*E* ≤ 1*e*–03) are shown. Genes previously characterized by in situ hybridization are indicated in bold. (*E*) Summarized expression of *MlNR1*, *MlNR2*, *MlBsh*, and *MlIslet* in the presumptive tentacle epithelium (TE), the invaginated tentacle bulb (TB), and near the apical organ (AO) during embryonic development. (*F*) Read counts for previously characterized transcription factors in tissues from adult *M. leidyi*. *MlNR1*, *MlBsh*, *MlIslet*, and *MlPRD10A* are significantly upregulated (≥2 log_2_-fold change) in the tentacle bulb (*MlNR1 P* = 0.004, *MlBsh P* = 0.010, *MlIslet P* < 0.001, *MlPRD10A P* = 0.026). MlNR2 is not differentially expressed (*P* = 0.893).

We further validated the colloblast and tentacle candidates by examining their expression in two adult tissues: tentacle bulbs and comb rows (fig. 4*A*). Over 70% of the candidate genes were also expressed in the adult tissues we sampled (*N* = 138/189 colloblast candidates, *N* = 130/165 tentacle candidates). Using differential expression analysis, we found that 33% of the colloblast candidates (*N* = 46/138) and 20% of the tentacle candidates (*N* = 26/130) were significantly upregulated in the tentacle bulb compared with the comb row (fig. 4*B*). Both sets of candidates were significantly enriched for tentacle bulb expression, compared with randomly selected data sets (*P* < 0.0001 for both).

Using a published data set reporting differential expression of genes across individual cell types in *M. leidyi* (Sebe-Pedros, Chomsky, et al. 2018), we found significant clustering of colloblast candidate genes (*N* = 18) in a single cell (C52; *P* < 0.0001) and another large cluster (*N* = 11) of colloblast candidates in a second cell (C53; fig. 4*C*). We also found significant clustering of tentacle candidates (*N* = 12) in a third cell (C54, *P* = 0.0015). The remaining expressed candidate genes were distributed across the other cell types, none of which had a cluster of more than four candidate genes. Cells C52, C53, and C54 were undescribed by Sebe-Pedros, Chomsky, et al. (2018); however, based on the significant overrepresentation of candidate genes in these cells, we suggest that C52 and C53 are colloblasts and C54 is another tentacle-specific cell type.

To characterize these putative colloblast and tentacle cell types further, we first searched both sets of candidates and all genes expressed in cells C52, C53, and C54 for transcription factors that have been previously characterized in *M. leidyi* (Pang and Martindale 2008; Jackson et al. 2010; Pang et al. 2010; Yamada et al. 2010; Pang et al. 2011; Reitzel et al. 2011; Schnitzler et al. 2012; Simmons et al. 2012; Schnitzler et al. 2014; Reitzel et al. 2016). Additionally, we performed reciprocal BLAST of these data sets against the human proteome and annotated the results of both searches using Gene Ontology (GO). To identify transcription factors, we searched specifically for the following GO terms: GO: 0003677—DNA binding; GO: 0003700—DNA binding, transcription factor activity; GO: 0006351—transcription, DNA templated; and GO: 0006355—regulation of transcription, DNA templated. These combined approaches led to the discovery of seventeen transcription factors (fig. 4*D*), five of which have been previously studied in *M. leidyi*: Nuclear Receptor 2 (*MlNR2*, ML305522a) from the colloblast candidate data set, Paired Class Homeobox 10a (*MlPRD10a*, ML148940a) from the tentacle candidate data set, Nuclear Receptor 1 (*MlNR1*, ML073417a) from cells C52 and C53, Brain Specific Homeobox (*MlBsh*, ML08269a) from cell C53, and the LIM Homeobox gene Islet (*MlIslet*, ML053012a) from cell C54 (Pang and Martindale 2008; Ryan et al. 2010; Reitzel et al. 2011; Simmons et al. 2012). Four of these (excluding MlPRD10a) have been previously characterized during embryonic development in *M. leidyi* using in situ hybridization. Whereas *MlNR2* is expressed ubiquitously throughout development, *MlNR1* is expressed in the tentacle bulb and apical organ, *MlBsh* is restricted to the tentacle bulb, and *MlIslet* is restricted to the apical organ (fig. 4*E*). Using tissue-specific transcriptomes from adults, we confirmed that *MlNR1*, *MlBsh*, *MlIslet*, and *MlPRD10a* are all upregulated in the tentacle bulb, relative to the comb rows (fig. 4*F*). *MlNR2* was expressed in both tentacle bulbs and comb rows but was not differentially expressed.

### Characterizing Candidate Genes

**Fig. 5.**
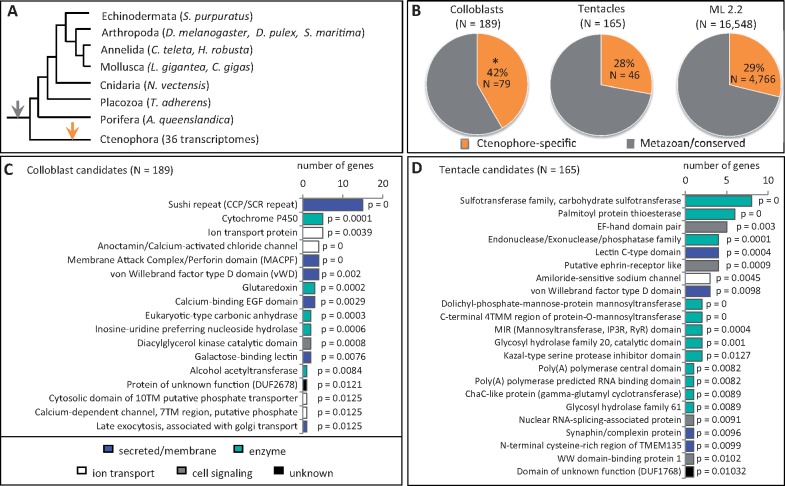
Ontology of candidate genes. (*A*) Cladogram of the taxa used to identify ctenophore-specific genes. Genes with significant BLAST hits (*E* ≤ 1*e*–02) to taxa other than Ctenophora are assumed to have been present in the common ancestor of all animals (grey arrow). Genes lacking significant hits to other metazoan taxa are assumed to have originated after ctenophores diverged from the rest of animals or to have been lost in the stem lineage giving rise to the rest of animals (orange arrow). (*B*) Ctenophore-specific genes (orange) are significantly enriched in the set of late-expressed colloblast candidates (*P* = 0.0007), but comprise similar proportions of the tentacle candidate genes and complete set of protein models from *Mnemiopsis leidyi* (ML 2.2). (*C*, *D*) Overrepresented annotations from colloblast and tentacle candidates, relative to ML2.2 protein model annotations.

Consistent with other studies of metazoan novelties (Johnson and Tsutsui 2011; Babonis et al. 2016), we hypothesized that the set of colloblast candidates would be enriched for novel (ctenophore-specific) genes. To test this, we used a reciprocal BLAST strategy to search candidate genes against a database of animal genomes (fig. 5*A*); we considered genes that lacked significant hits outside of Ctenophora (*E* ≥ 1*e*-02) to be ctenophore-specific. Over 40% (79/189) of the colloblast candidates were ctenophore-specific, whereas only 28% (46/165) of the tentacle candidates and 29% (4,766/16,548) of all protein models (ML2.2) were ctenophore-specific (fig. 5*B*). Random sampling confirmed that colloblast candidates were significantly enriched for novel genes (*P* < 0.0001) whereas tentacle candidates were not (*P* = 0.643).

To evaluate their putative function, we annotated both sets of candidate genes against the InterPro Consortium database using Interproscan (Jones et al. 2014). We found that 61% (116/189) of the colloblast candidates and 76% (125/165) of the tentacle candidates were annotated (supplementary file 1, Supplementary Material online). We further identified 17 GO terms that were significantly overrepresented in colloblast candidates (fig. 5*C*) and 22 GO terms significantly overrepresented in tentacle candidates (fig. 5*D*). Among colloblast candidates, overrepresented categories were largely associated with secretion/cell membrane recognition (e.g., sushi/SCR, MACPF, vWD, Ca-EGF, lectin, golgi transport) and enzymes involved in cellular metabolism (e.g., cytochrome P450, glutaredoxin, carbonic anhydrase, nucleoside hydrolase, acetyltransferase). Among tentacle candidates, the largest overrepresented category consisted of enzymes involved in posttranslational modification (i.e., sulfotransferase, thioesterase, phosphatase, mannosyltransferase, glycosyl hydrolase, cyclotransferase).

### Searching for Ctenophore Adhesive Proteins

We used BLAST to search candidate genes against a set of known adhesive proteins from other invertebrates (Hennebert et al. 2015). (Sequences provided in supplementary file 2, Supplementary Material online.) Five colloblast candidates and four tentacle candidates had significant hits to adhesive proteins (*E* ≤ 1*e*–03), yet each of these genes had better hits to other proteins in the Uniprot database (www.uniprot.org; table 1). Next, we compared protein family (Pfam) domains from the known adhesives to domains identified from candidate genes using Interproscan. From the 48 confirmed adhesive proteins, we identified 17 Pfam domains. One domain was shared among all three data sets (von Willebrand factor type D domain, PF00094), one was shared by adhesives and colloblasts only (EGF-like calcium-binding domain, PF07645), and one was shared by adhesives and tentacles (Chitin binding domain, PF01607). Consistent with our BLAST results, neither colloblast- nor tentacle candidates exhibited significant overlap with Pfam domains from known adhesives (colloblasts *P* = 0.5008, tentacles *P* = 0.4459).

**Table 1.** Putative Adhesive Genes from Colloblast (C) and Tentacle (T) Data Sets. Only Significant Hits (≤1*E*–03) are Shown.

Set	ML Gene ID	Top hit—Adhesives	*E*-Value	Top Hit—Uniprot	*E*-Value
C	ML020113a^a^	ABR68008.1 matrilin-like 85 kDa protein (*Ambigolimax valentianus*)	6.16*E*–11	O89103|C1QR1_MOUSE Complement component C1q receptor (*Mus musculus*)	1.37*e*–20
C	ML056914a	ABR68008.1 matrilin-like 85 kDa protein (*Ambigolimax valentianus*)	3.12 *E*–10	Q14246|AGRE1_HUMAN Adhesion G protein-coupled receptor E1 (*Homo sapiens*)	5.55*e*–25
C	ML50011a	ABR68008.1 matrilin-like 85 kDa protein (*Ambigolimax valentianus*)	2.35 *E*–08	O08999|LTBP2_MOUSE Latent-transforming growth factor beta-binding protein 2 (*Mus musculus*)	7.16*e*–24
C	ML223525a^a^	AHN92641.1 sea star footprint protein 1 (*Asterias rubens*)	3.04 *E*–06	Q9HC84|MUC5B_HUMAN Mucin-5B (*Homo sapiens*)	1.91e–15
C	ML32223a^a^	AHN92641.1 sea star footprint protein 1 (*Asterias rubens*)	1.19 *E*–06	Q9Y493|ZAN_HUMAN Zonadhesin (*Homo sapiens*)	8.27*e*–16
T	ML14246a	AHN92641.1 sea star footprint protein 1 (*Asterias rubens*)	1.15 *E*–12	Q02817|MUC2_HUMAN Mucin-2 (*Homo sapiens*)	1.22*e*–17
T	ML14247a^a^	AHN92641.1 sea star footprint protein 1 (*Asterias rubens*)	3.07 *E*–12	Q9HC84|MUC5B_HUMAN Mucin-5B (*Homo sapiens*)	3.79*e*–17
T	ML154123a^a^	AHN92641.1 sea star footprint protein 1 (*Asterias rubens*)	8.48 *E*–09	Q02817|MUC2_HUMAN Mucin-2 (*Homo sapiens*)	3.51*e*–13
T	ML056959a	AFP57565.1 aggregate gland silk factor 1, partial (*Latrodectus hesperus*)	1.34 *E*–10	A2VD00|EIF3A_XENLA Eukaryotic translation initiation factor 3 subunit A (*Xenopus laevis*)	2.72*e*–22

Note.—Candidate genes were searched using BLAST against a database of empirically derived adhesive genes concatenated with the complete Uniprot database.

a Indicates presence of signal peptide. ML gene IDs refer to ML2.2 (https://research.nhgri.nih.gov/mnemiopsis/).

**Fig. 6.**
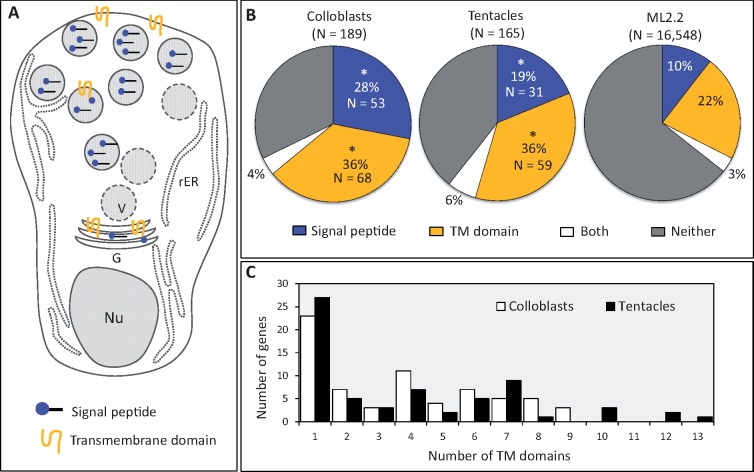
Identification of features associated with subcellular localization. (*A*) Diagram of an immature colloblast after Storch and Lehnert-Moritz (1974) showing developing secretory vesicles. (*B*) Both colloblast and tentacle candidates were significantly enriched for genes encoding signal peptides (blue) and transmembrane domains (yellow) (*P* < 0.0001 for all). (*C*) Number of genes encoding single- and multi-pass transmembrane domains from colloblast and tentacle candidates.

Given that we did not find strong BLAST support for the homology of candidate genes and proteins from other biological adhesives, we searched instead for features known to be enriched among described adhesive proteins, including: secretion signal peptide, single-pass transmembrane domains, and regions of low sequence complexity (Waite et al. 2005; Endrizzi and Stewart 2009). Using SignalP (Petersen et al. 2011) and TMHMM (Krogh et al. 2001), we found that 28% (53/189) of the colloblast candidates encoded a signal peptide and 36% (68/189) encoded one or more transmembrane domains (fig. 6*A* and *B*). By comparison, 19% (31/165) of tentacle candidates encoded a signal peptide and 36% (59/165) encoded transmembrane domains. Both sets of candidate genes were significantly enriched for signal peptides and transmembrane domains, relative to random sets drawn from ML2.2 (*P* < 0.0001, for both). The number of transmembrane domains varied from 1 to 9 among colloblast candidates and from 1 to 13 among tentacle candidates (fig. 6*C*), although the number of transmembrane domains in these candidate gene sets did not differ significantly from samples drawn randomly from ML2.2 (colloblasts *P* = 0.5961, tentacles *P* = 0.4756). Therefore, neither set of candidate genes was enriched for single-pass transmembrane domains. Finally, we assessed sequences in both data sets for regions of low complexity using the program Segmasker (Wootton 1994). Contrary to our expectations based on other biological adhesives, colloblast and tentacle candidates were not enriched for regions of low-complexity (colloblasts *P* = 0.427, tentacles *P* = 0.98).

### Searching for Ctenophore Toxin Proteins

Moss et al. (2001) suggested the possibility that colloblasts or other secretory cells in ctenophores may secrete a toxin. To test this hypothesis, we used BLAST to search both sets of candidate genes against a database of known animal venoms/toxins, referred to hereafter as “ToxProt” (Jungo et al. 2012). From the colloblast candidates, we identified a single gene (ML263512a) with a significant match in the ToxProt database (*E* ≤ 1*e*–03); however, this gene had better hits to Uniprot proteins outside of the ToxProt database (table 2). Among tentacle candidates, we identified 12 sequences with significant hits in the ToxProt database, only one of which (ML435831a) had an equivalent/better hit to a protein in the ToxProt database than to any nontoxin proteins in the Uniprot database.

**Table 2.** Putative toxin Genes from Colloblast (C) and Tentacle (T) Data Sets. Only Significant Hits (*E* ≤ 1*e*–03) are Shown.

Set	ML Gene ID	Top Hit—ToxProt	*E*-Value	Top Hit—Uniprot	*E*-Value
C	ML263512a	Q25338|LITD_LATTR Delta-latroinsectotoxin-Lt1a *Latrodectus tredecimguttatus* (Mediterranean black widow spider)	5.37*E*–07	G5E8K5|ANK3_MOUSE Ankyrin-3 *Mus musculus* (mouse)	3.32*e*–12
T	ML01511a	Q3SB11|CALGL_TROCA Calglandulin *Tropidechis carinatus* (Australian rough-scaled snake)	3.68*E*–12	P02595|CALM_PATSP Calmodulin *Patinopecten* sp. (scallop)	1.19*e*–24
T	ML01571a	Q3SB11|CALGL_TROCA Calglandulin *Tropidechis carinatus* (Australian rough-scaled snake)	3.52*E*–27	P24044|CALM_PLAFA Calmodulin *Plasmodium falciparum*	7.35*e*–39
T	ML01786a	Q66S03|LECG_THANI Galactose-specific lectin nattectin *Thalassophryne nattereri* (Copper Joe toadfish)	5.93*E*–07	P82596|PLC_HALLA Perlucin *Haliotis laevigata* (Smooth Australian abalone)	2.17*e*–09
T	ML056959a	Q66S03|LECG_THANI Galactose-specific lectin nattectin *Thalassophryne nattereri* (Copper Joe toadfish)	6.35*E*–05	A2VD00|EIF3A_XENLA Eukaryotic translation initiation factor 3 subunit A *Xenopus laevis* (African clawed frog)	2.73*e*–22
T	ML056965a	A3FM55|LECM1_HYDHA C-type lectin 1 *Hydrophis hardwickii* (Hardwick’s spine-bellied seasnake)	2.97*E*–05	P21328|RTJK_DROME RNA-directed DNA polymerase from mobile element jockey *Drosophila melanogaster* (fruit fly)	1.98*e*–16
T	ML070216a	Q3SB11|CALGL_TROCA Calglandulin *Tropidechis carinatus* (Australian rough-scaled snake)	2.87*E*–13	P25071|CML12_ARATH Calmodulin-like protein 12 *Arabidopsis thaliana* (Mouse-ear cress)	5.02*e*–30
T	ML075218a	Q02989|LITA_LATTR Alpha-latroinsectotoxin-Lt1a *Latrodectus tredecimguttatus* (Mediterranean black widow spider)	5.69*E*–06	P16157|ANK1_HUMAN Ankyrin-1 *Homo sapiens* (human)	3.50*e*–10
T	ML14246a	P0DKM9|TU11_LOPAL Turripeptide OL11-like *Lophiotoma albina* (Sea snail)	2.72*E*–04	Q02817|MUC2_HUMAN Mucin-2 *Homo sapiens* (human)	1.22*e*–17
T	ML273210a	Q8AY75|CALGL_BOTIN Calglandulin *Bothrops insularis* (Golden lancehead snake)	5.69*E*–25	P27164|CALM3_PETHY Calmodulin-related protein *Petunia hybrida* (Petunia)	3.94*e*–56
T	ML35385a	G0LXV8|LATA_LATHA Alpha-latrotoxin-Lh1a *Latrodectus hasseltii* (Redback spider)	1.14*E*–06	Q8Q0U0|Y045_METMA Putative ankyrin repeat protein MM_0045 *Methanosarcina mazei* (anaerobic archaeobacter)	4.38*e*–14
T	**ML435831a**^a^^,^^b^	**P58912|TX60B_PHYSE DELTA-alicitoxin-Pse2b *Phyllodiscus semoni* (Night anemone)**	**1.20*E*–20**	**P58912|TX60B_PHYSE DELTA-alicitoxin-Pse2b *Phyllodiscus semoni* (Night anemone)**	**1.20*e*–20**
T	ML45397a	Q9XZC0|LCTA_LATTR Alpha-latrocrustotoxin-Lt1a *Latrodectus tredecimguttatus* (Mediterranean black widow spider)	1.37*E*–10	Q5ZLC8|ANR52_CHICK Serine/threonine-protein phosphatase 6 regulatory ankyrin repeat subunit C *Gallus gallus* (chicken)	1.90*e*–14

Note.—Candidate genes were searched using BLAST against a database of known animal venom/toxin genes (ToxProt) concatenated with the complete Uniprot database.

a Indicates presence of signal peptide.

b indicates an equivalent or better hit in the ToxProt database. ML gene IDs refer to ML2.2 (https://research.nhgri.nih.gov/mnemiopsis/).

We then compared Pfam domains from the ToxProt database with domains from the colloblast and tentacle data sets. From the 6,665 genes in ToxProt, we identified 174 Pfam domains. Four domains were shared among all three data sets: Ankyrin repeat-containing domain (PF12796), Membrane attack complex component/perforin (MACPF) domain (PF01823), Thrombospondin type-1 (TSP1) repeat (PF00090), and Thyroglobulin type-1 (PF00086); three domains were shared between ToxProt and colloblasts only: Beta-propeller repeat TECPR (PF06462), DNA/RNA nonspecific endonuclease (PF01223), Immunoglobulin domain (PF13927); and five domains were shared between ToxProt and tentacles only: Ankyrin repeat (PF00023, PF13637), C-type lectin-like (PF00059), EF-hand domain (PF13499, PF13833), Kazal domain (PF00050, PF07648), and ShK domain (PF01549). Contrary to our expectations, neither set of candidates exhibited significant overlap with ToxProt Pfam domains (colloblasts *P* = 0.7987, tentacles *P* = 0.0873).

Finally, we searched the suite of genes identified from cells C52, C53, and C54 (Sebe-Pedros, Chomsky, et al. 2018) against the ToxProt database to determine if there was additional support for the secretion of toxins in these putative tentacle cell types. We identified five cells with significant clusters of ToxProt genes: C17 (*P* = 0.016), C21 (*P* = 0.036), C25 (*P* = 0.037), C47 (*P* = 0.029), and C54 (*P* = 0.020; supplementary fig. 1, Supplementary Material online). Cell C54 was identified by the authors only by the presence of a protein with an ShK domain, a domain originally identified from sea anemone toxins.

### A Common Origin for Colloblasts and Neurons

**Fig. 7.**
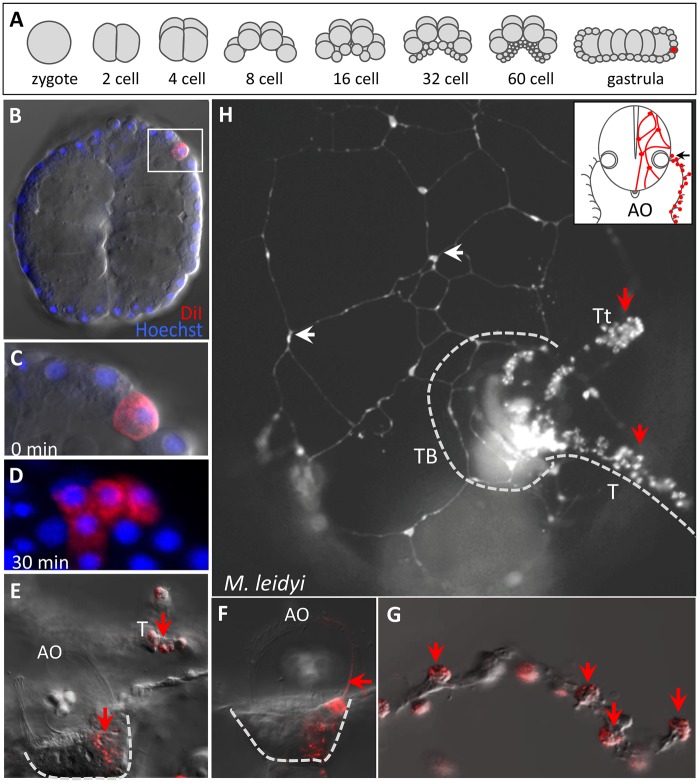
Neurons and colloblasts share a common progenitor. (*A*) Embryonic cleavage stages in *Mnemiopsis leidyi*. Random micromeres (red) were injected at the late gastrula stage. (*B*) Optical section of a live gastrula immediately following injection of DiI (red); nuclei are labeled with Hoechst. (*C*) High magnification image of the boxed area in B showing a single injected micromere. (*D*) High magnification image of a clone of labeled cells 30 min after DiI injection. (*E*–*G*) A live cydippid larva with two labeled populations of cells: in the floor plate of the apical organ (AO) and the colloblasts of the tentacle (T). (*E*) Both cell populations are depicted in a single focal plane. (*F*) A different focal plane showing labeling of cells and dome cilia (arrow) on one side of the apical organ. (*G*) High magnification image of the extended tentacle; colloblasts (arrows) are the only labeled cells in the tentacle. (*H*) Cydippid larva with DiI-labeled neurons of the subepidermal nerve net in the body wall (white arrows) and colloblasts (red arrows); the animal is viewed from the tentacular plane (black arrow) as summarized in the inset. TB—tentacle bulb, T—tentacle, Tt—tentilla.

Although cell fate has been fairly well characterized in *M. leidyi* (Martindale and Henry 1997; Martindale and Henry 1999; Henry and Martindale 2001), previous studies of cell fate have been performed only up to the 60-cell (pregastrula) stage; as such, little is known about the fate of cells differentiating at later stages of development. To characterize the developmental origin of the colloblasts in *M. leidyi*, we randomly labeled single cells in the vicinity of the presumptive tentacle epithelium in late gastrula stage embryos with a fluorescent dye (DiI; fig. 7*A*–*D*) and allowed embryos to develop to the cydippid stage, as previously described. From 28 embryos with individually labeled cells, we recovered seven cydippids (25%) with labeled colloblasts on the side corresponding to the injected micromere. Surprisingly, all seven of these cydippids also exhibited DiI-labeled neurons, either in the floor of the apical organ (fig. 7*E*–*G*) or in the peripheral nerve net (fig. 7*H*), suggesting that colloblasts and neurons differentiate from a common progenitor that acquires its identity after gastrulation.

### No Common Origin for Colloblasts and Cnidocytes

We tested the hypothesis that colloblasts and cnidocytes share a common evolutionary origin by searching for orthologous genes in these two cell types. Using OrthoFinder, we generated orthology groups using protein models from *M. leidyi* and the sea anemone *Nematostella vectensis.* From this analysis, we identified four groups containing at least one candidate gene (colloblast or tentacle) and at least one of the proteins identified as cnidocyte-specific in a recent study using single-cell sequencing from *N. vectensis* (Sebe-Pedros, Saudemont, et al. 2018; table 3).

**Table 3.** Orthology Groups Containing Colloblast (C) or Tentacle (T) Candidates from *M. leidyi* and Cnidocyte Genes from *N. vectensis*.

Set	ML Gene ID	NV Gene ID	Others in Group	Description
C	ML020113a, ML056914a, ML50011a	NVJ_2203	ML282520a, NVJ_108241, NVJ_113453, NVJ_117150, NVJ_117297, NVJ_119340, NVJ_123710, NVJ_129169, NVJ_137797, NVJ_142234, NVJ_146869, NVJ_154796, NVJ_157742, NVJ_16432, NVJ_198567, NVJ_202189, NVJ_208146, NVJ_209642, NVJ_210066, NVJ_223762, NVJ_224641, NVJ_22881, NVJ_2483, NVJ_3250, NVJ_32913, NVJ_37776, NVJ_46752, NVJ_48353, NVJ_61301, NVJ_67572, NVJ_6789, NVJ_70073, NVJ_79239, NVJ_79524, NVJ_80132, NVJ_80370, NVJ_83827, NVJ_84687, NVJ_87211, NVJ_87454, NVJ_89626, NVJ_9760, NVJ_99210	Fibrillin; Latent-transforming growth factor beta-binding protein 4
C	ML305522a	NVJ_165424	NVJ_101676, NVJ_108851, NVJ_114090, NVJ_132075, NVJ_134436, NVJ_167880, NVJ_169225, NVJ_183874, NVJ_189134, NVJ_203423, NVJ_209681, NVJ_242271, NVJ_89471, NVJ_93844, NVJ_94673, NVJ_99425	Nuclear receptor; Retinoic acid receptor RXR-gamma-B
T	ML00965a	NVJ_99284, NVJ_175881		Protein O-mannosyl-transferase 2
T	ML10468a, ML435831a	NVJ_200058	ML41821a, ML020060a, NVJ_109596, NVJ_1099, NVJ_1115, NVJ_1173, NVJ_166322, NVJ_196985, NVJ_205444, NVJ_211816	DELTA-alicitoxin/sea anemone venom protein

Note.—Cnidocyte genes were extracted from Sebe-Pedros, Saudemont, et al. 2018. ML gene IDs refer to ML2.2 (https://research.nhgri.nih.gov/mnemiopsis/). NV gene ID refers to the *N. vectensis* genome (https://genome.jgi.doe.gov/Nemve1/Nemve1.home.html, last accessed September 10, 2018).

Two groups included colloblast candidates and cnidocyte genes; the first group contains orthologs of fibrillin, a glycoprotein component of the extracellular matrix, and the second contains orthologs of retinoic acid receptors (RxRs). Upon closer inspection, the colloblast candidate in this latter group turned out to be the previously studied nuclear receptor *MlNR2* (Reitzel et al. 2011). We used BLAST to search *MlNR2* against *B. ovata* and confirmed that this gene is missing from both the transcriptome and the genome of *B. ovata*. The other two groups contained tentacle candidates and cnidocyte genes. The first of these contained orthologs of protein-O-mannosyl transferase 2, an important regulator of protein glycosylation. Genes in the second group share homology with DELTA-alicitoxin, a pore-forming toxin from sea anemones. Compared with data sets sampled randomly from ML2.2, orthology groups containing cnidocyte genes were not significantly enriched for colloblast (*P* = 0.8519) or tentacle (*P* = 0.7869) candidates.

## Discussion

First described nearly 200 years ago (Eschscholtz 1829), ctenophores remain a poorly understood group of animals. By combining phylogeny, natural variation in morphology, analyses of embryonic and adult gene expression, and detailed sequence annotations, we have identified and characterized genes associated with tentacle-specific cell types. While we recognize the possibility that our data sets may include genes not associated with these traits, the overrepresentation of late-expressed genes (fig. 3*C*) with numerous peaks in expression (fig. 3*D* and *E*), significant upregulation in the adult tentacle bulb (fig. 4*B*), and significant clustering in specific isolated cell types (fig. 4*C*), suggests this approach was effective for identifying genes associated with tentacle cell identity.

As part of this work, we have likely uncovered novel components of an undescribed biological adhesive. Consistent with other adhesives, colloblast candidate genes were enriched for domains associated with secretion, membrane recognition, and subcellular protein trafficking (fig. 5, supplementary file 1, Supplementary Material online). Furthermore, colloblast candidates were enriched for a hydrophobic N-terminal signal peptide (fig. 6). Signal peptides are important for directing proteins to the vesicles in numerous secretory cell types including cnidocytes (Anderluh et al. 2000), cells from venom glands (Jones et al. 1992), and adhesive-secreting cells from other animal groups (Hennebert et al. 2015). Thus, the genes we identified as colloblast candidates are consistent with the genes expected to be expressed in a cell undergoing synthesis, packaging, and storage of secreted proteins.

Surprisingly, we found no BLAST support for the homology of colloblast candidates with other biological adhesive proteins (table 1) and, unlike other biological adhesives, colloblast candidates were not enriched for regions of low-complexity. Combined with the overrepresentation of ctenophore-specific genes among colloblast candidates (fig. 5), our results suggest that the origin of the colloblast adhesive was largely independent from the evolution of adhesives in other biological systems. Unlike other animal adhesives (e.g., sea star foot protein, mussel byssal threads), the colloblast adhesive must be fast-acting (“instantaneous”) but need not be permanent (Flammang et al. 2009) and these constraints may have facilitated the origin of an adhesive with unique properties in the stem ctenophore. Indeed, we suggest that rapid evolution of existing genes (Martin-Duran et al. 2017), resulting in *de novo* acquisitions of novel peptide motifs may have promoted the origin of the colloblast adhesive.

We further leveraged the secondary loss of tentacles in the genus *Beroe* to identify compelling candidate genes for future studies of other tentacle specific cell types in ctenophores. Tentacle candidates were enriched for signal peptides as well as enzymes involved in posttranslational protein modification (figs. 5 and 6, supplementary file 1, Supplementary Material online). One intriguing interpretation is that these enzyme-rich tentacle secretory cells are some type of gland cell engaged in the production and secretion of a ctenophore toxin. In support of this, we identified one gene from the tentacle candidates (ML435831a) that encodes both a signal peptide and a MACPF domain, and appears to be an ortholog of actinoporin, a pore-forming DELTA-alicitoxin found in sea anemone cnidocytes (table 3; Oshiro et al. 2004; Rachamim et al. 2015). Further supporting the potential role of this tentacle cell type in producing a toxin, we demonstrate significant clustering of tentacle candidates (fig. 4*F*) in a single cell (C54) that also expresses the largest number of genes with significant hits in the ToxProt database (supplementary fig. 1, Supplementary Material online). While empirical observations are essential for evaluating the function of this cell type, these results suggest that ctenophores may incapacitate their prey by secretion of pore-forming toxins from a tentacle specific gland cell. A toxin-secreting cell may have provided many ecological benefits, even among taxa lacking colloblasts, which could explain why this cell type may have been retained in *Haeckelia*.

Notably, both data sets (colloblast and tentacle candidate genes) were largely devoid of transcription factors. Essential for activating and/or repressing the expression of effector genes (e.g., secreted or structural products), transcription factors are known to be highly pleiotropic, regulating gene expression in numerous regulatory networks. *Sox* genes, for example, are likely involved in tentacle morphogenesis based on their expression in the tentacle bulb of both *M. leidyi* and *Pleurobrachia bachei* (Jager et al. 2008; Schnitzler et al. 2014); however, the fact that each *Sox* gene is expressed in additional domains outside of the tentacle bulbs in both species suggests these genes play many roles in the development of ctenophores. Consistent with this, *Sox* genes were not identified among the colloblast or tentacle candidates and the transcriptome of *B. ovata* encodes complete orthologs of all six ctenophore *Sox* genes (supplementary file 3, Supplementary Material online).

Annotation of both the candidate gene data sets and the putative colloblast (C52, C53) and tentacle (C54) cell types published previously (Sebe-Pedros, Chomsky, et al. 2018) enabled us to identify seventeen putative transcription factors that may play a role in patterning tentacle-specific cell types in *M. leidyi* (fig. 4*D*–*F*). Possible colloblast transcription factors (*MlNR1*, *MlNR2*, and *MlBsh*) are all known to be expressed in the tentacle bulb during tentacle morphogenesis (Pang and Martindale 2008; Reitzel et al. 2011) and we demonstrate significant upregulation of *MlNR1* and *MlBsh* in the adult tentacle bulb as well. The role of the putative toxin cell transcription factors (*MlIslet* and *MlPRD10a*) is not as clear. While both are upregulated in the adult tentacle bulb, *MlIslet* does not appear to be expressed in the tentacle primordia during embryonic development (Simmons et al. 2012) and the spatial expression of *MlPRD10a* has not been characterized (Ryan et al. 2010). Intriguingly, the genome of *B. ovata* encodes clear orthologs of *MlNR1*, *MlBsh*, and *MlIslet*, but lacks orthologs of *MlNR2* and *MlPRD10a*. Given that *MlNR2* and *MlPRD10a* were identified as candidate genes from our phylogenetic analysis, we propose that knockdown of these genes in *M. leidyi* should result in loss of colloblasts and other tentacle-specific secretory cells.

Surprisingly, our data suggest a common embryological origin for colloblasts and neurons, as both cell lineages appear to be the descendants of a single micromere labeled in the late gastrula stage in *M. leidyi* (fig. 7). Previous cell lineage studies performed at earlier stages of development found that neural and epidermal cells arose from a common precursor (Martindale and Henry 1997; Martindale and Henry 1999). Our results extend these observations, showing that epidermal cells differentiate from this common lineage before the separation of the neuronal and colloblast identities, as only the latter two cell types arose from micromeres labeled at later stages of development. This confirms a closer embryological relationship of the latter two cell types. Assuming that neurons are homologous across ctenophores (Hernandez-Nicaise 1973), these results imply that the loss of colloblasts resulted from disruption of the colloblast-specific branch of this lineage, independent of the segregation of neurons. Considering that ctenophores in the genus *Haeckelia* have tentacle bulbs and tentacles but lack colloblasts, we further suggest that the loss of colloblasts was independent of the development of the tentacle bulb. Additional studies of cell fate during embryogenesis in ctenophores with and without tentacle bulbs would shed much needed light on the evolution of morphological diversity in this group.

**Fig. 8.**
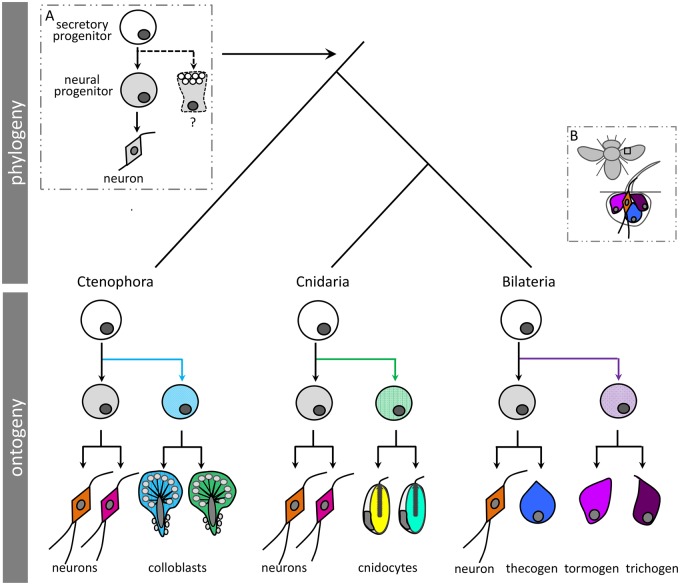
A possible scenario for the origin of secretory cell diversity in animals. (*A*) The common ancestor to ctenophores, cnidarians, and bilaterians may have already had a secretory cell progenitor (white) giving rise to neurons and some other unspecialized secretory cell. This pathway may have been independently co-opted (colored lines) to give rise to novel secretory cell types in ctenophores, cnidarians, and bilaterians. (*B*) The common origin of the neural and secretory cells responsible for making the sensory apparatus in flies supports this hypothesis in bilaterians. Sponges and placozoans have been excluded for simplicity.

The shared embryological origin of colloblasts and neurons underscores one striking commonality between colloblasts and cnidocytes, as both cell types differentiate from a progenitor cell that also gives rise to neurons (Richards and Rentzsch 2014; Flici et al. 2017). Importantly, however, we found no additional evidence of a shared origin for these two cell types. Indeed, we found that colloblasts and cnidocytes express largely unique suites of genes as only four orthology groups were identified from among the hundreds of colloblast and cnidocyte candidates (table 3). Thus, rather than inferring the origin of some ancestral colloblast/cnidocyte prototype, we suggest that these novel secretory cell types arose independently in each lineage by co-option of a progenitor cell that already had the capacity for regulated cell secretion (fig. 8). Assuming nervous systems are homologous across animals (Jekely et al. 2015; Ryan and Chiodin 2015), it is likely that this progenitor cell already gave rise to neurons and possibly other secretory cell types in the ancestor to the lineage encompassing ctenophores, cnidarians, and bilaterians (inset A, fig. 8). Studies characterizing the development of the epidermal sensory organs (sensilla) in flies support this explanation for the origin of novel secretory cells in bilaterians as well, since both the neural and secretory cells (thecogen, tormogen, and trichogen cells) underlying the sensilla also differentiate from a common progenitor (inset B; Hartenstein and Posakony 1989).

The relationship of specialized animal secretory cells to neurons suggests that there may be some underlying property of “neural” progenitor cells that makes them more likely to give rise to novel cell types. Because of their critical role in cell–cell communication, neurons have a phenotype that enables the packaging, storage, and delayed secretion of their products. It is possible that this pathway is easy to co-opt for other secretory functions, which could explain why multiple independent lineages of novel cell types seem to have evolved from a progenitor giving rise to neurons. Alternatively, cells that secrete a novel product may simply be easy to positively identify as novel cell types, artificially inflating the relationship of neurons to novelty. Considering *Sox* genes are expressed in the common progenitor of neurons and cnidocytes in cnidarians (Richards and Rentzsch 2014) and in the tentacle bulb of ctenophores (Jager et al. 2008; Schnitzler et al. 2014), we suggest that *Sox* genes may be good candidates for conferring general secretory cell identity across metazoans. Understanding the origin of other types of secretory cells (e.g., gland cells) in ctenophores and cnidarians and characterizing their developmental relationship to colloblasts/cnidocytes and neurons will be important for further assessing the ubiquity of this relationship between *Sox* gene expression and secretory cell phenotype.

The candidate genes described here now form the basis of future investigations into the origin, differentiation, and development of colloblasts and other tentacle-specific cell types in ctenophores. Future studies aimed at constructing the regulatory networks underlying ctenophore secretory cells (including neurons, colloblasts, and gland cells) will provide a unique opportunity to simultaneously characterize the poorly understood nervous system of ctenophores and probe the process by which novel secretory cells evolve. Cells with novel functions can be important for facilitating expansion into new ecological niches, ultimately promoting speciation and diversification. Over evolutionary time, *Beroe* and *Haeckelia* have transitioned to prey types (other ctenophores and cnidarians, respectively) that are atypical for ctenophores, suggesting trophic specialization and evolutionary loss of cell types may have facilitated diversification in Ctenophora.

## Materials and Methods

### Animal Collection, Tissue Processing, and Transcriptome Assembly

Most specimens were collected during blue-water dives or using remotely operated-underwater vehicles from a region of the Eastern Central Pacific near the Monterey Bay Aquarium Research Institute (Moss Landing, CA), as described previously (Francis et al. 2015). These samples were snap frozen in liquid nitrogen and sequenced using a paired-end sequencing protocol at the University of Utah on an Illumina HiSeq 2000 platform with 100 amplification cycles. Briefly, read order was randomized and low-quality reads, adapters, and repeats were removed. For efficiency, subsets of reads were used to assemble transcriptomes. Assembly was performed with both Velvet/Oases v1.2.09/0.2.08 (Zerbino and Birney 2008; Schulz et al. 2012) and Trinity r2012-10-05 (Grabherr et al. 2011). Transcripts from both assemblers were combined and redundant sequences were removed using the sequniq utility in the GenomeTools package (Gremme et al. 2013).

For the developmental transcriptome series, adult *M. leidyi* were collected from the estuary behind the University of Florida’s Whitney Laboratory for Marine Bioscience (St. Augustine, FL) and maintained in the dark for 8 h to induce spawning. Zygotes were collected before first cleavage (time 0) and embryos were collected every 30–60 min for the first 20 h of development. Embryos were collected individually (*N* = 3–6 embryos per time) and snap frozen on dry ice. Samples were prepared and sequenced on an Illumina HiSeq 2500, as described previously (Levin et al. 2016). *B. ovata* was collected from a public boat ramp on the Intracoastal Waterway in Port Orange, FL. Adults were spawned in the lab following the protocol for *M. leidyi* and embryos were collected individually (*N* = 4 per collection time) at 0, 6, 10, and 20 h post fertilization. RNA was extracted from all 16 embryos and from 4 adults and sent to the Genomic Sequencing and Analysis Facility at the University of Texas, Austin, for library preparation and sequencing on an Illumina HiSeq 2500.

For validation of putative colloblast and tentacle genes, we assembled tissue-specific transcriptomes from adult *M. leidyi* collected from the estuary behind the Whitney Lab. Tissues (tentacle bulbs and comb rows) were freshly isolated from wild caught animals and snap frozen on dry ice. RNA extraction, library preparation, and sequencing were performed by the Interdisciplinary Center for Biotechnology Research at the University of Florida. Three independent replicates of each tissue were sequenced on a single lane of a HiSeq 3000 using a paired-end protocol. Differential expression analysis was performed using DESeq2 v1.20.0 (Love et al. 2014) in R v3.5.0 (R Core Development Team, 2008). Transcripts with ≥2 log_2_-fold change and an adjusted *P*-value ≤ 0.05 were considered differentially expressed. Raw sequence data have been deposited in the European Nucleotide Archive (accession PRJEB28334).

### Phylogenetics/18S Tree

We aligned 18S sequences from 36 ctenophores using MAFFT with default parameters (Nakamura et al. 2018). Trees were generated using three approaches: IQ-TREE v1.5.5 (Nguyen et al. 2015), RAxML (Stamatakis 2014) with 10 maximum parsimony starting trees, and RAxML with 10 random starting trees. A likelihood value for each tree was generated using RAxML; the tree produced by IQ-TREE had the highest likelihood value. We used AfterPhylo v0.9.1 (https://github.com/qiyunzhu/AfterPhylo; last accessed September 10, 2018) to create a tree that collapsed all branches with <50% bootstrap support.

### Identification and Annotation of Candidate Genes

To identify genes that had been lost in the lineage of ctenophores lacking colloblasts (*Beroe* + *Haeckelia*), we first created orthologous gene groups for the complete transcriptomes of all 36 species of ctenophore using OrthoFinder v1.1.8 (Emms and Kelly 2015). Colloblast candidates were genes present in orthology groups containing ≥70% of the taxa (including *M. leidyi*) but were missing from all three species of *Beroe* (*B. ovata*, *B. forskalii*, and *B. abyssicola*) and from both species of *Haeckelia* (*H. rubra* and *H. beehleri*). Requiring these genes to be present in at least 70% of the transcriptomes (rather than 100%) allowed us to account for stochasticity in gene expression (i.e., genes not expressed at the time the animal was collected) and for gene losses that did not affect the maintenance of colloblasts. The number of candidate genes we recover varies considerably when we allow this cutoff to range from 50% to 100%, but there was no clear choice for the single best proportion to use (supplementary fig. 2, Supplementary Material online). We arbitrarily chose 70% but FASTA files of candidate genes recovered for all other cutoffs are provided in the GitHub Repository for this publication: https://github.com/josephryan/2018-Babonis_et_al_Ryan. Tentacle candidates were present in orthology groups containing ≥70% of the taxa (including *M. leidyi* and at least one species of *Haeckelia*) but lacking any species of *Beroe*. Three character states were possible for genes expressed in *Haeckelia* (see dagger, fig. 3*A*): present in both species, present in *H. rubra* only, or present in *H. beehleri* only. In each case, *Haeckelia* was counted toward the 70% total required to constitute ubiquitous expression across ctenophores.

### Temporal Expression of Candidate Genes in *M. leidyi* Embryos

We examined the expression of candidate genes during embryonic development in *M. leidyi* using stage-specific RNA-Seq data (NCBI GEO accessions GSE60478 and GSE111748). First, we removed colloblast and tentacle candidate genes with no expression (*N* = 7/189 colloblast candidates and *N* = 10/165 tentacle candidates with TPM = 0 at all time points). We also removed sequences with no expression from the ML2.2 (https://research.nhgri.nih.gov/mnemiopsis/) protein models (*N* = 3, 902/16, 548 genes with TPM = 0 at all time points). We then compared ratios of late gene expression (after the onset of tentacle morphogenesis; 12–20 hpf) to early gene expression (0–9 hpf). Late-expressed genes were those with a ratio >1. We searched the set of gene models (ML2.2) using the same approach. We used QT clustering (Heyer et al. 1999) to cluster candidate genes with similar expression patterns.

### Cell Specific Expression of Candidate Genes

We examined the distribution of candidate genes across individual cells from adult *M. leidyi* isolated for single cell sequencing by Sebe-Pedros, Chomsky, et al. (2018). Significant clustering of candidate genes in individual cells was assessed with 10,000 random draws of similarly sized data sets.

### Identifying Ctenophore-Specific Genes

To test the hypothesis that phylum-specific cell types are enriched in novel proteins (encoded by phylum-specific genes), we first examined the colloblast and tentacle data sets for ctenophore-specific genes using alien_index (https://github.com/josephryan/alien_index). In brief, this method uses a reciprocal BLAST strategy to identify taxon-specific genes as those which have sufficiently poor matches (*E* > 1*e*–02) to taxa outside Ctenophora. Genomes for nonctenophore metazoan taxa examined in this study were downloaded from EnsemblMetazoa on January 21, 2016 and consist of the following: *Amphimedon queenslandica* (Porifera), *Capitella teleta* (Annelida), *Crassostrea gigas* (Mollusca), *Daphnia pulex* (Arthropoda), *Drosophila melanogaster* (Arthropoda), *Helobdella robusta* (Annelida), *Lottia gigantea* (Mollusca), *N. vectensis* (Cnidaria), *Strigamia maritima* (Arthropoda), *Strongylocentrotus purpuratus* (Echinodermata), *Trichoplax adhaerens* (Placozoa). Genes that lacked significant hits in this database of animal taxa were considered ctenophore-specific. We also used Interproscan v5.26-65.0 (Jones et al. 2014) to annotate candidate genes against the InterPro Consortium database using the default settings. These analyses were used to evaluate the percentage of candidate genes with annotations. GO annotations had previously been assigned to *M. leidyi* gene models (ML2.2) using Trinotate (Levin et al. 2016).

### Presence of Signal Peptides and Transmembrane Domains in Target Genes

We searched candidate genes for signal peptides using SignalP v4.1 (Petersen et al. 2011) and for transmembrane domains using TMHMM v2.0 (Krogh et al. 2001). Generally, the genome of *M. leidyi* encodes fewer signal peptides and transmembrane domains than does the human genome (supplementary fig. 3, Supplementary Material online); however, this may simply reflect the fact that ctenophore sequences were not included in the training set for the SignalP and TMHMM algorithms. To test if the number of signal peptides and transmembrane domains identified by SignalP and TMHMM in our candidate gene data sets was greater than random chance, we built 10,000 randomly assembled size-matched data sets from ML2.2 (*N* = 189 for colloblast candidates and *N* = 165 for tentacle candidates). We then ran SignalP on these random sets to determine how many searches produced more signal peptides and transmembrane domains than our initial search.

### Amino Acid Composition and Low Complexity Sequences

We determined the composition of amino acids in the colloblast candidate and tentacle candidate data set and compared them to 10,000 randomly assembled size-matched data sets. To determine if these candidate data sets had high numbers of low-complexity sequence stretches, we used Segmasker v1.0.0 (Wootton 1994) to identify regions of low complexity in these data sets as well as the random data sets.

### Sequence Similarity to Known Adhesive- and Toxin-Related Proteins

To identify putative adhesive genes, we used BLASTP v2.5.0 (Altschul et al. 1990) to search candidates against the Uniprot database concatenated with the 48 adhesive proteins reported previously (Hennebert et al. 2015). To identify venoms/toxins, we searched candidates against the ToxProt database, a Uniprot database annotated for known venom/toxin genes using the Animal Toxin Annotation Project (www.uniprot.org/program/Toxins). The Uniprot database was downloaded on October 13, 2017 and the ToxProt database was downloaded on September 5, 2017. Hits with *E* ≤ 1*e*–03 were considered significant.

### Domain Similarity between Candidates and Known Adhesive- and Toxin-Related Proteins

We used the Interproscan results to test whether our sets of candidate genes disproportionally shared Pfam domains with proteins in the Adhesives and ToxProt databases. Towards this, we compared the number of domains shared by the candidate genes and 10,000 randomly assembled size-matched data sets drawn from each database.

### Cnidocyte Orthology Analysis

Using OrthoFinder (as above) we grouped ML2.2 with the complete set of protein models from *N. vectensis* downloaded from JGI (https://genome.jgi.doe.gov/Nemve1/Nemve1.home.html), and a subset of proteins identified by Sebe-Pedros, Saudemont, et al. (2018) as cnidocyte specific but not found in JGI (www.cnidariangenomes.org/download/nve.gene_models.vie130208). We then searched for orthology groups containing at least one candidate (colloblast or tentacle) from *M. leidyi* and at least one cnidocyte-specific protein. We assessed significance by searching for shared orthology groups in 10,000 randomly assembled size-matched groups drawn from ML2.2 and the NVJ database augmented with additional cnidocyte-specific sequences.

### Cell Lineage Tracing in *M. leidyi*

Experiments were performed as described previously (Martindale and Henry 1997; Martindale and Henry 1999). Briefly, individual micromeres of gastrula stage embryos were microinjected with saturated DiI (DiIC18(3); Molecular Probes, OR, USA) prepared in soybean oil. Embryos were either imaged immediately or reared to the cydippid stage in 0.2 μm filtered seawater at room temperature before imaging.

### Statistics and Code Availability

We used a Monte Carlo approach to assess the significance of our observations. Briefly, we randomly selected 10,000 data sets each of size *N* = 189 genes or size *N* = 165 genes from *M. leidyi* gene models (ML2.2) and compared the distribution of these random draws to colloblast and tentacle candidates, respectively. This approach was used to detect enrichment of late-expressed genes, ctenophore-specific genes, GO annotations, shared Pfam domains, signal peptides, transmembrane domains, regions of low sequence complexity, clustering in individual cells, and clustering with cnidocyte orthologs. Scripts and files for these analyses are in the GitHub Repository for this publication: https://github.com/josephryan/2018-Babonis_et_al_Ryan.

## Supplementary Material

Supplementary data are available at *Molecular Biology and Evolution* online.

## Supplementary Material

Supplementary Data
