# Supplementary material for: Integrating Embryonic Development and Evolutionary History to Characterize Tentacle-Specific Cell Types in a Ctenophore
Source: Mol Biol Evol. 2018 Aug 30;35(12):2940–56. doi: 10.1093/molbev/msy171 (PMC6278862; doi:10.1093/molbev/msy171)
Supplement: Supplementary Data [file msy171_supp.zip › Supplementary_File_4_Figures.pdf]

Title: Integrating embryonic development and evolutionary history to characterize the colloblast, a ctenophore-specific cell type

Authors: Leslie S. Babonis, Melissa B. DeBiasse, Warren R. Francis, Lynne M. Christianson, Anthony G. Moss, Steven H. D. Haddock, Mark Q. Martindale, and Joseph F. Ryan

Supplementary Figures S1- S3

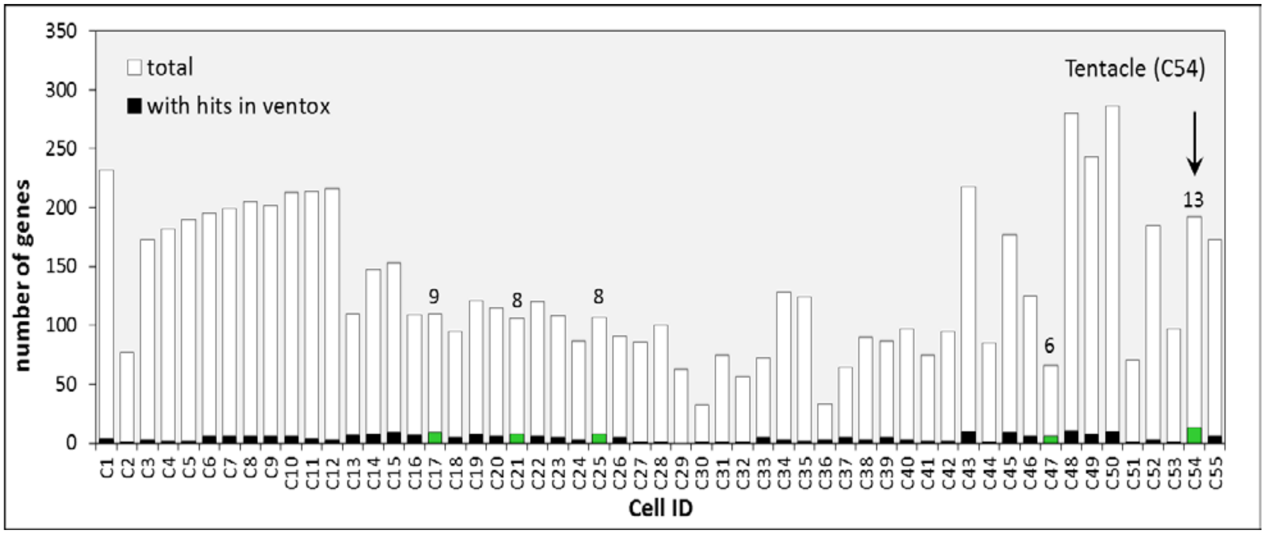

Figure S1. The number of genes expressed in each cell type identified by Sebe-Pedros et al., 2018a (open bars) and the number of genes from each cell type with significant hits (E < 1e-03) against the ToxProt (www.uniprot.org/program/Toxins) database (filled bars). Cells with significant clusters of genes with significant hits in ToxProt are colored green (C17 p=0.016, C21 p=0.0356, C25 p=0.0367, C47 p=0.0289, C54 p=0.0204). C54 (putative tentacle cell type) had the largest cluster of ToxProt genes (N = 13). Gene IDs and BLAST hits for these 13 genes are provided in Supplementary File 1 (tables). Code for statistical analysis is provided in the GitHub repository associated with this study (https://github.com/josephryan/2018-Babonis_et_al_Ryan.)

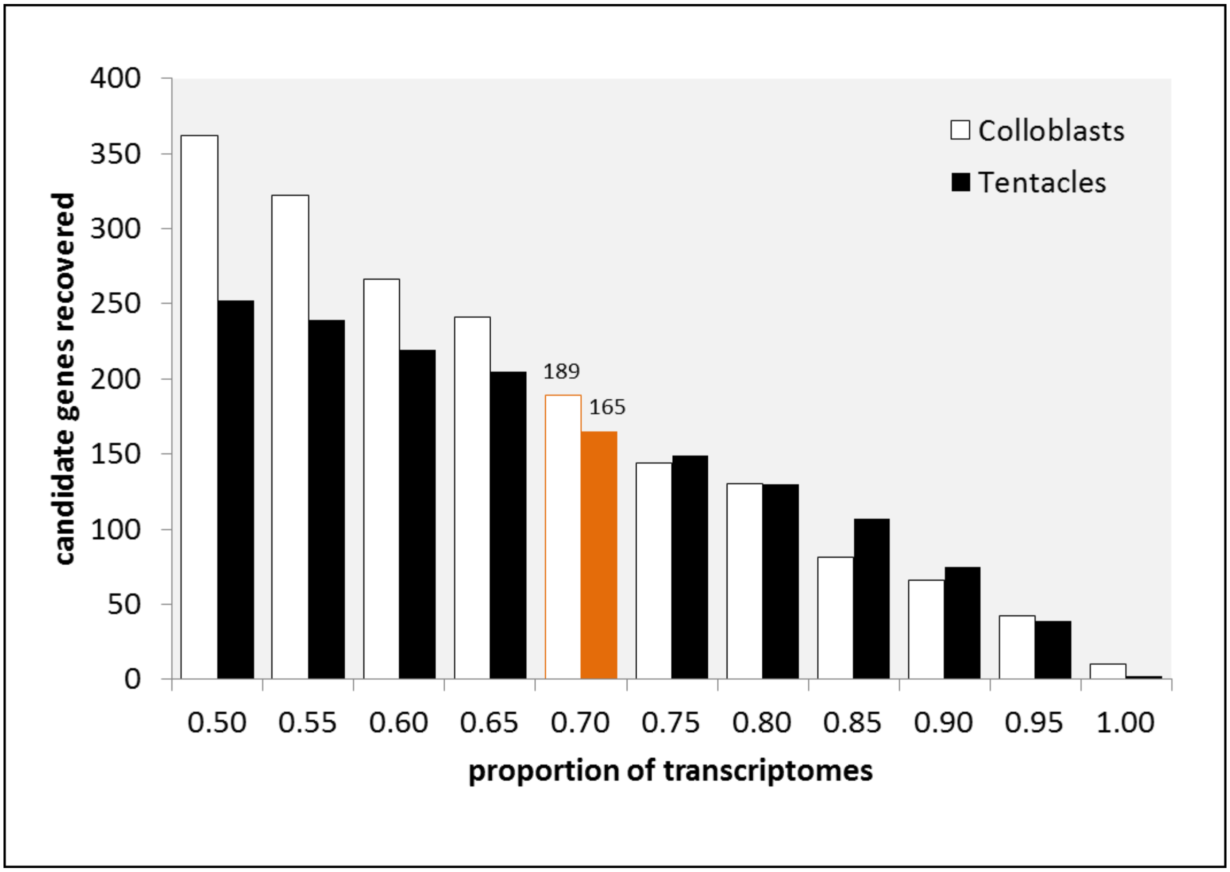

Figure S2. The number of candidate genes recovered increases as the proportion of taxa required to express an ortholog varies from 0.50 to 1.0. In this study, tentacle candidates (N = 165) were genes that had orthologs in 70% of the study taxa but were specifically missing from the genus *Beroe*. Likewise, colloblast candidates (N = 189) had orthologs in 70% of the taxa but were missing from *Beroe* and *Haeckelia*. See Methods for additional details. As there was no clear way to choose a cutoff to use, we chose 0.70. FASTA files of candidate genes for all other cutoffs are provided in the GitHub repository associated with this study (https://github.com/josephryan/2018-Babonis_et_al_Ryan.)

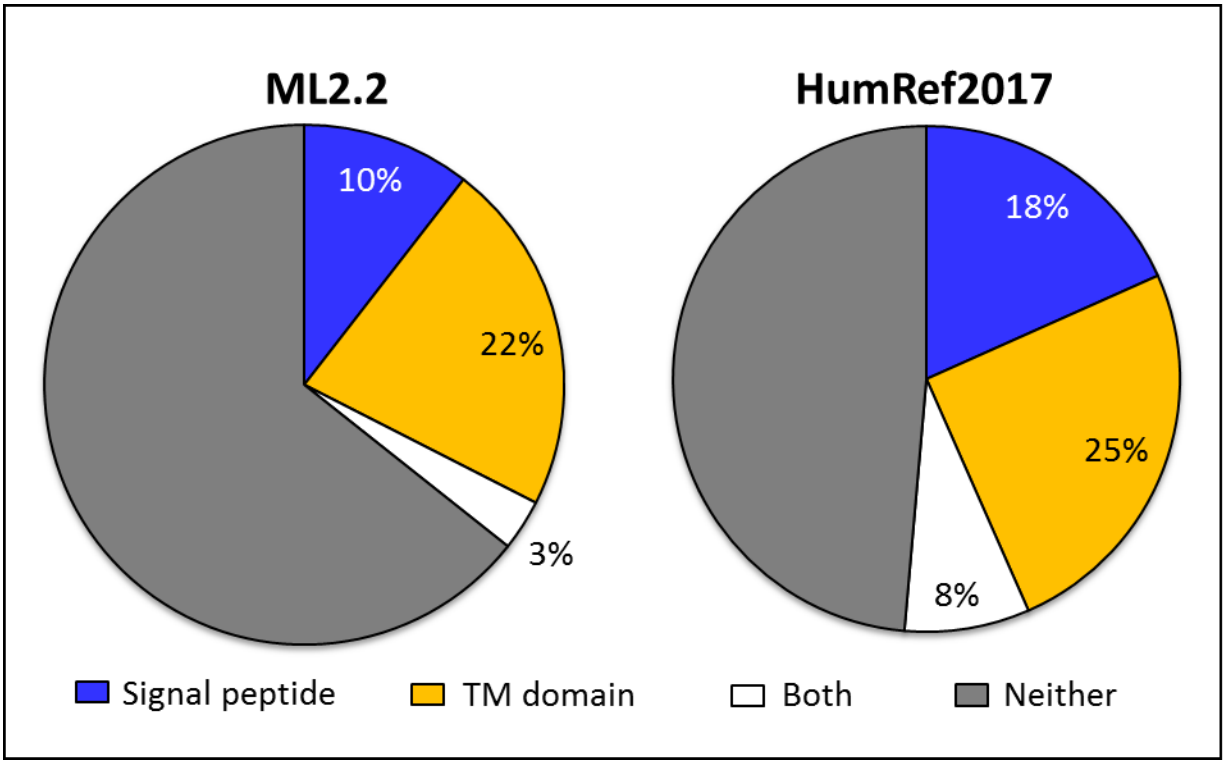

Figure S3. Comparison of sequences containing signal peptides, transmembrane (TM) domains, or both in *Mnemiopsis leidyi* (ML2.2) and human (HumRef2017).
